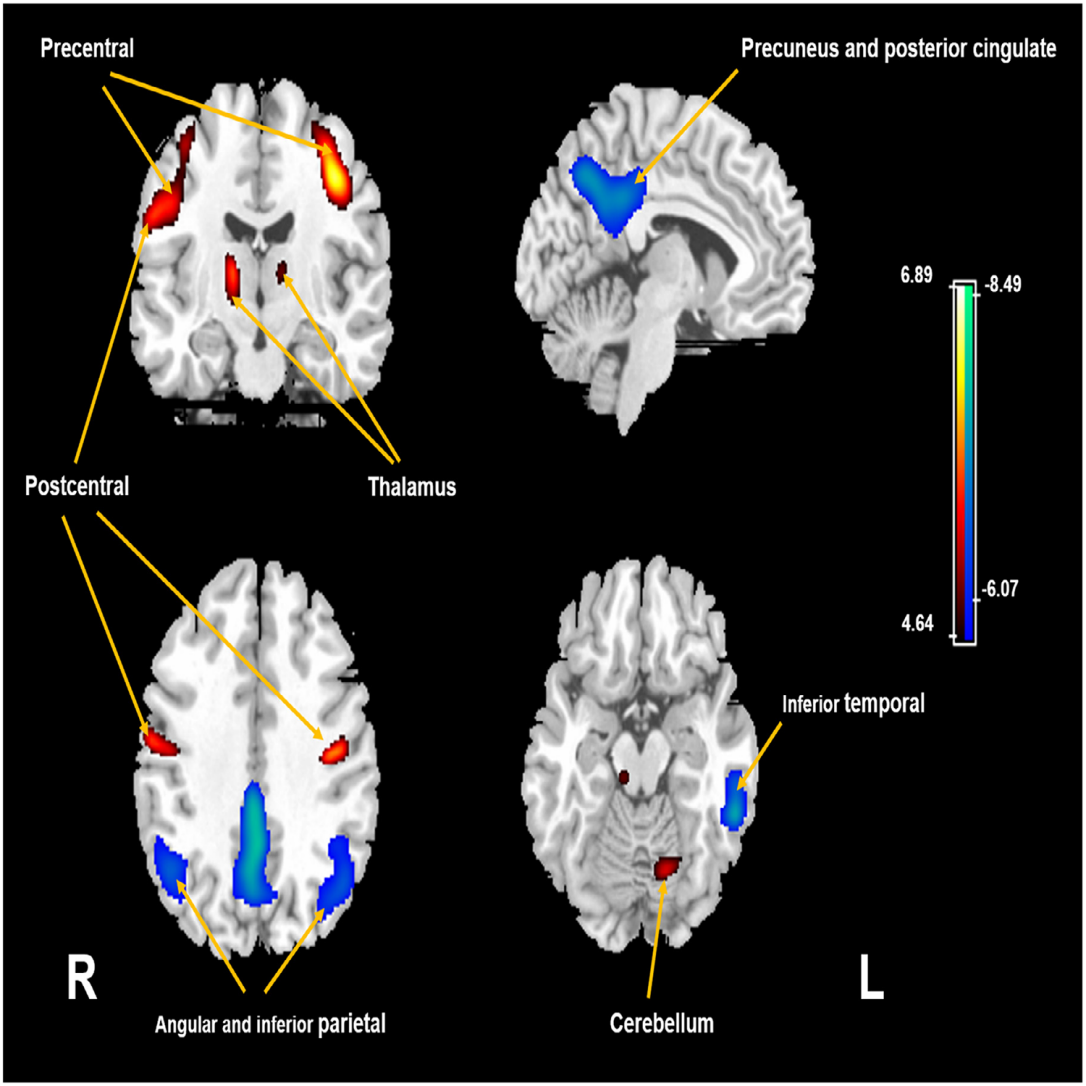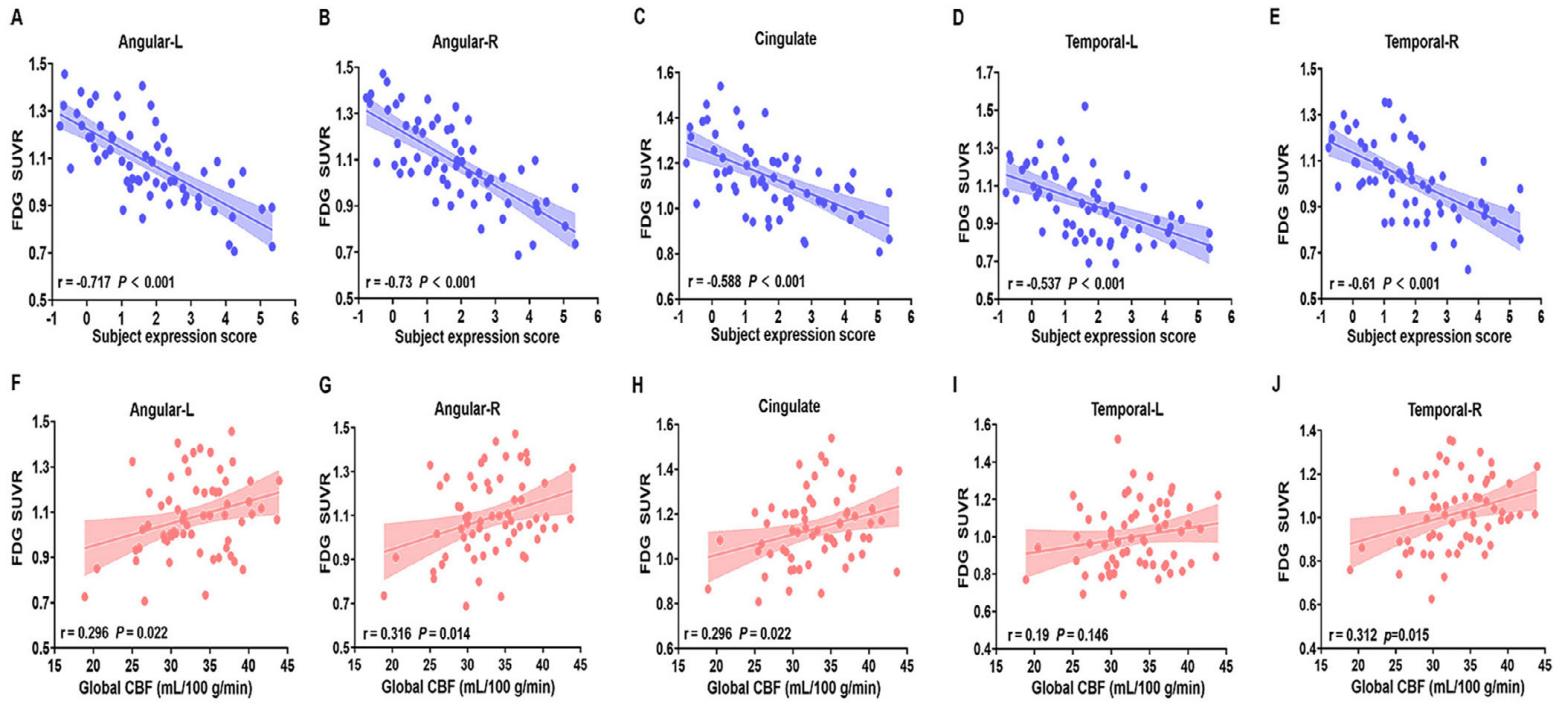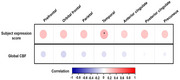# Cerebral perfusion is correlated with cerebral metabolism and amyloid deposition in Alzheimer's disease

**DOI:** 10.1002/alz70855_099218

**Published:** 2026-01-05

**Authors:** Ping Che, Nan Zhang

**Affiliations:** ^1^ Tianjin Medical University General Hospital, Jin Tian, Jin Tian, China

## Abstract

**Background:**

Cerebral blood flow (CBF) changes play a pivotal role in the pathogenesis and progression of Alzheimer's disease (AD), but their effects on other pathological processes, such as neurodegeneration and amyloid‐β deposition, are unclear. We investigated the correlations between cerebral perfusion measured with arterial spinal labeling (ASL) and cerebral metabolism and amyloid deposition on positron emission tomography (PET) scans in AD

**Method:**

Sixty‐four AD patients and 56 cognitively unimpaired controls were included. Cerebral perfusion was indicated by the expression of AD‐related perfusion pattern (ADRP), global CBF and the relative value of regional CBF. The standardized uptake value ratio (SUVR) of regions of interest (ROIs) was calculated for ^18^F‐fluorodeoxyglucose (FDG)‐PET and ^11^C‐Pittsburgh Compound B (PiB)‐PET images in AD.

**Result:**

The subject expression score of ADRP showed strong negative correlations with FDG SUVR in all ROIs and a positive correlation with PiB SUVR in temporal. FDG SUVR in some ROIs were also positively correlated with global CBF and relatively regional CBF in the temporoparietal cortex, precuneus and posterior cingulate, and negatively correlated with relatively regional CBF in the thalamus and pre‐ and postcentral regions. The PiB SUVR of the ROIs were negatively correlated with the relatively regional CBF in the left inferior parietal region and were positively correlated with the relatively regional CBF in the right thalamus and left precentral regions.

**Conclusion:**

CBF was significantly correlated with hypometabolism and amyloid deposition in AD.